# Antimicrobial Activity of Two Different Types of Silver Nanoparticles against Wide Range of Pathogenic Bacteria

**DOI:** 10.3390/nano14020137

**Published:** 2024-01-07

**Authors:** Viktoriia Holubnycha, Yevheniia Husak, Viktoriia Korniienko, Svetlana Bolshanina, Olesia Tveresovska, Petro Myronov, Marharyta Holubnycha, Anna Butsyk, Thomas Borén, Rafal Banasiuk, Arunas Ramanavicius, Maksym Pogorielov

**Affiliations:** 1 Medical Institute, Sumy State University, 2, Rymskogo-Korsakova St., 40007 Sumy, Ukraine; e.gusak@med.sumdu.edu.ua (Y.H.); viktoriia.korniienko@lu.lv (V.K.); s.bolshanina@chem.sumdu.edu.ua (S.B.); a.pereshyvailo@med.sumdu.edu.ua (O.T.); p.myronov@med.sumdu.edu.ua (P.M.); marharyta.holubnycha@student.sumdu.edu.ua (M.H.); maksym.pogorielov@lu.lv (M.P.); 2 Faculty of Chemistry, Silesian University of Technology, 44-100 Gliwice, Poland; 3 Institute of Atomic Physics and Spectroscopy, University of Latvia, 3 Jelgavas St., LV-1004 Riga, Latvia; 4 Department Medical Biochemistry and Biophysics, Umeå University, SE-901 87 Umeå, Sweden; anna.butsyk@umu.se (A.B.); thomas.boren@umu.se (T.B.); 5 NanoWave, 02-676 Warsaw, Poland; rafal.banasiuk@nanopure.pl; 6 Mechanical Faculty, Gdańsk University of Technology, G. Narutowicza 11/12, 80-233 Gdańsk, Poland; 7 Department of Physical Chemistry, Institute of Chemistry, Faculty of Chemistry and Geosciences, Vilnius University, Naugarduko Str. 24, LT-03225 Vilnius, Lithuania

**Keywords:** silver nanoparticles, ESKAPE pathogens, antimicrobial activity, antibiofilm activity

## Abstract

The emergence of antibiotic-resistant bacteria, particularly the most hazardous pathogens, namely *Enterococcus faecium*, *Staphylococcus aureus*, *Klebsiella pneumoniae*, *Acinetobacter baumannii*, *Pseudomonas aeruginosa*, and *Enterobacter* spp. (ESKAPE)-pathogens pose a significant threat to global health. Current antimicrobial therapies, including those targeting biofilms, have shown limited effectiveness against these superbugs. Nanoparticles, specifically silver nanoparticles (AgNPs), have emerged as a promising alternative for combating bacterial infections. In this study, two types of AgNPs with different physic-chemical properties were evaluated for their antimicrobial and antibiofilm activities against clinical ESKAPE strains. Two types of silver nanoparticles were assessed: spherical silver nanoparticles (AgNPs-1) and cubic-shaped silver nanoparticles (AgNPs-2). AgNPs-2, characterized by a cubic shape and higher surface-area-to-volume ratio, exhibited superior antimicrobial activity compared to spherical AgNPs-1. Both types of AgNPs demonstrated the ability to inhibit biofilm formation and disrupt established biofilms, leading to membrane damage and reduced viability of the bacteria. These findings highlight the potential of AgNPs as effective antibacterial agents against ESKAPE pathogens, emphasizing the importance of nanoparticle characteristics in determining their antimicrobial properties. Further research is warranted to explore the underlying mechanisms and optimize nanoparticle-based therapies for the management of infections caused by antibiotic-resistant bacteria.

## 1. Introduction

Our attitude to antibiotics has been transformed from “magic pill” to “carefully prescribed medicine” since their discovery. A high number of antibiotics lost their effectiveness due to the formation of drug resistance within several years after creation. These days, pharmaceutical companies decrease the interest in new antibiotics investigation due to high costs and the duration of the research with unpredictable financial and clinical results [1]. To tackle antibiotic resistance, WHO created a list of priority pathogens, which requires the development of new antimicrobials [2]. It includes ‘ESKAPE’ pathogens such as *Enterococcus faecium*, *Staphylococcus aureus*, *Klebsiella pneumoniae*, *Acinetobacter baumannii*, *Pseudomonas aeruginosa*, and *Enterobacter* spp.). Most of them are multidrug-resistant isolates, which is one of the greatest challenges in clinical practice. It significantly increases hospital stay, cost of care, and mortality. Moreover, ESKAPE pathogens are the leading cause of nosocomial infections throughout the world [3]. The formation of antimicrobial resistance in ESKAPE pathogens is associated with the modification of drugs, changes in bacteria cells, and biofilm formation [4]. ESKAPE pathogens possess a high ability to colonize various surfaces and form biofilms, which are substantial features of antimicrobial-resistant strains [5]. The formation of biofilm consists of several steps, starting with attachment. Bacterial cells can recognize and attach to specific proteins, promoting bacterial colonization. From this point, the biofilm grows by the formation of a matrix of extracellular polymeric substances (EPS) around bacterial cells [6]. The ESKAPE pathogens provide cell–cell communication via a quorum sensing system to control the genes that modulate drug resistance and pathogenic behaviors [7]. All listed mechanisms provide bacteria in biofilm a 10- to 1000-fold higher resistance to antibiotic treatment [8]. Consequently, searching for new therapeutic approaches for the management of the infections caused by ESKAPE pathogens is crucial.

The combination of antibiotics with adjuvants, bacteriophages, antimicrobial peptides, and photodynamic light therapy has been tested to control infection caused by ESKAPE pathogens [9]. Unfortunately, these strategies have not yet shown greater effectiveness [10]. Nanomaterials are considered promising alternatives to existing antimicrobial agents [11]. Metallic nanoparticles (NPs) possess many unique features [12]. Studies over the past decades have provided important information on the use of silver NPs [13], zinc oxide NPs [14], copper NPs [15], composite Cu2ZnSnS4 monograins [16], composite Pd/MoS2/Ti-based coatings [17] for the killing of pathogenic microbes. Silver nanoparticles show both antibacterial and anti-biofilm activities [18], unlike antibiotics. The antimicrobial effect of AgNPs is complex and includes several pathways that prevent the formation of antimicrobial resistance [19,20]. In addition, silver nanoparticles could be used for the delivery of other antimicrobial substances. However, wide clinical application of the silver nanoparticles is limited due to their potential cytotoxicity and lack of complex studies.

Previous research has demonstrated that the physical and chemical parameters of the nanoparticles and the method of their synthesis influence their antimicrobial activity [21]. Some studies suggest that smaller particle sizes, higher surface-area-to-volume ratios [22], and a particular shape of nanoparticles are associated with higher antibacterial effectiveness. Another important parameter of NPs is Zeta potential, which explains the stability, dispersion, and surface charge of the nanoparticles. Zeta potential greater than +30 mV or less than −30 mV indicates high stability of nanoparticles in dry powder form [23].

Chemical synthesis is the most widely used method of AgNP production. The essential components are a salt precursor, a reducing agent, and a stabilizer. They influence the nanoparticle parameters and reduce drawbacks [24]. Previous studies have demonstrated the important role of polymer-based capping agents in controlling the size and shape of AgNPs [25]. It was shown that PVP-coated AgNPs were more stable, less toxic, and had better antimicrobial activity compared to AgNPs stabilized with other chemicals [26]. However, the influence of these parameters on the effectiveness of AgNPs against microorganisms is limited. 

The study of NPs’ antibacterial mechanisms stays on the priority list for scientists. Several cross-sectional studies suggest following antimicrobial mechanisms as oxidative stress, metal ion release, and non-oxidative mechanisms [27]. However, the generalizability of much-published research on this issue is problematic, and the nature of NPs’ antibacterial mechanisms remains unclear. Aside from this, several studies have recognized the effectiveness of AgNPs against individual ESKAPE pathogens [28,29], but there is no single comparative investigation of silver nanoparticles’ effect on clinical strains of all ESKAPE pathogens in one study.

The aim of this study was to identify the most important factors influencing the antibacterial and antibiofilm effectiveness of two different types of AgNPs against clinical ESKAPE pathogens.

## 2. Materials and Methods

### 2.1. Silver Nanoparticles’ Physical and Chemical Parameters

Two different types of silver NPs were used in this study. AgNPs-1 were synthesized at Sumy State University with the polyol method and characterized earlier [30]. Briefly, the mixture of ethylene glycol (40 mL) and PVP (6.8 g) was heated to 155 °C for 30 min. Then, AgNO3 (0.68 g) was added to ethylene glycol (40 mL) and stirred at room temperature. Then, it was added dropwise to the preliminary heated mixture, and the synthesis reaction was allowed to proceed for 1 h, constantly stirring with a magnetic stirrer. The sample was washed three times by re-dispersion in isopropanol (1:1) and centrifugation. The precipitate was dried in a vacuum dryer and then diluted in sterile distilled water. AgNPs-2 were provided using Nano Pure Co., Warsaw, Poland. AgNO_3_, PVP, and NaClO were mixed in borosilicate bottles. The reaction was performed in an aqueous environment and utilizes light emitted by commercially available 1 W light-emitting diodes (λ = 420 nm) as the catalyst. A detailed description of AgNPs-2 synthesis and their structural and chemical parameters is represented in [31]. 

**Figure 1:**
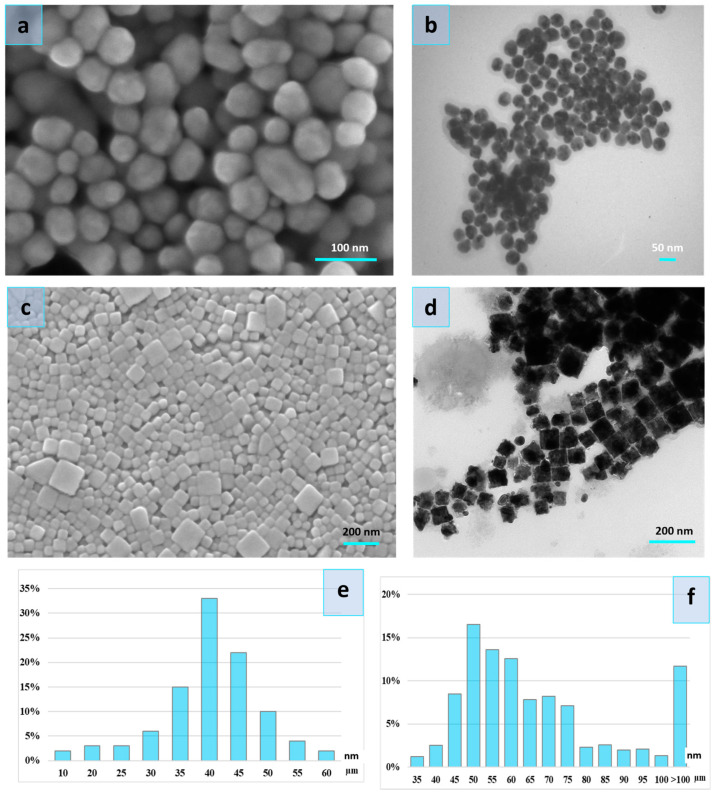
Scanning electron microscopy image of the AgNPs-1 (**a**) and AgNPs-2 (**c**), Transmission electron microscopy image of the AgNPs-1 (**b**) and AgNPs-2 (**d**), and particle size distribution of the AgNPs-1 (**e**) and AgNPs-2 (**f**).

To evaluate the influence of AgNP morphological parameters on antimicrobial activity, we characterized them using both scanning electron microscopy (SEM) and transmission electron microscopy (TEM) at the Umeå Core Facility for Electron Microscopy (UCEM), Umeå, Sweden; (Figure 1c) SEM microscope Carl Zeiss Merlin FESEM (GmbH, Hamburg, Germany); (Figure 1d) TEM microscope Talos L120C (Thermo Fisher Scientific, Waltham, MA, USA), ImageJ 1.50c. software (https://imagej.nih.gov/ij/) (Version 1.5, University of Wisconsin, Madison, WI, USA). Based on size measurement, the surface area to volume ratio (SA:V) was calculated by Equations for (1) cube, (2) sphere:

(1) $$\frac{6a^{2}}{a^{3}}=\frac{6}{a}$$

(2) $$\frac{{4\pi r}^{2}}{\frac{4}{3}{\pi r}^{3}}=\frac{3}{r}$$

*a*—length of the cube side of NPs, *r*—radius of the sphere NPs.

To assess the stability, dispersion, and surface charge of the nanoparticles and their influence on the antibacterial activity of the nanoparticles, we defined the average value of Zeta potential (AZP). The zeta potential of nanoparticle suspension was evaluated with a Malvern zetasizer nano ZS at room temperature.

To estimate the influence of silver ions release on the antibacterial activity of nanoparticles, the stock solution of AgNPs-1 and Ag-NPs-2 were diluted with distilled water up to 129.0 µg/mL and 194.7 µg/mL, respectively. For the separation of Ag+ ions from AgNPs, 10 mL of a solution containing silver nanoparticles was centrifuged in a centrifuge (8000 rpm) for 5 min. Concentrations of silver ions in the initial solutions of nanoparticles and in the supernatants formed after centrifugation of these solutions for 2 days were measured using the atomic absorption spectroscopy method (AAS) using an S-115 M1 spectrophotometer. The parameters of analysis were the following: a lamp with a hollow cathode for silver (λ (Ag) = 328.1 nm), an oxidizing flame is a mixture of propane-butane-air gases.

### 2.2. Bacterial Strains and Culture Conditions

ESKAPE pathogens (*Enterococcus faecium*, *Staphylococcus aureus*, *Klebsiella pneumoniae*, *Acinetobacter baumannii*, *Pseudomonas aeruginosa*, *Enterobacter* spp.) were isolated from patients with respiratory infections. Identification of the bacterial strains was performed by examining their morphological, staining, biochemical, and antigenic features. Microorganisms were tested on sensitivity to beta-lactams, glycopeptides, *cephalosporins*, aminoglycosides, fluoroquinolones, and azalides. The strains resistant to at least one antimicrobial drug in three or more antimicrobial categories were selected as multi-resistant and stored at −80 °C for further research. This study protocol was approved by the Institutional Ethics Committee (Sumy State University, Protocol 7/15 from 18 November 2020) after the informed consent collection from the patients. 

Media (Mueller-Hinton broth and agar) for the cultivation of microorganisms were purchased at Hi Media (Maharashtra, India). Sigma Aldrich (St. Louis, MO, USA) provided gentian violet and resazurin.

### 2.3. Antimicrobial Activity of AgNPs

Minimum inhibitory concentration (MIC) was determined using the broth microdilution method established by CLSI [32] using Mueller-Hinton broth. Briefly, overnight cultures were diluted in Mueller-Hinton broth to a concentration of 5 × 10^5^ CFU/mL per well. A volume of 180 µL of each diluted bacterial suspension was dispensed into a flat-bottom polystyrene 96-well plate, and then a serially diluted stock solution of the AgNPs (20 µL) was added into each well to reach the concentrations in the range 0.31–200 µg/mL. Wells without AgNPs and without bacteria were set up as positive and negative controls, respectively. Plates were incubated at 37 °C for 24 h. The assay was performed in triplicate. MIC was established based on the wells with the lowest concentration of the investigated samples that completely inhibit the visual growth of bacteria (no turbidity). 

A time-kill assay was performed to determine the killing kinetics of stationary-phase bacteria. The overnight culture of ESKAPE strains was diluted to 5 × 10^5^ CFU/mL in Mueller-Hinton broth. Silver nanoparticles were added in triplicates at concentrations equal to 1 MIC for every strain. Bacteria were incubated aerobically at 37 °C. Aliquots from each well (10 µL) were taken in 1, 3, 6, 12, 24 h and inoculated onto Mueller-Hinton agar. CFUs were counted after the incubation of plates at 37 °C for 24 h. This involved calculating all visible colonies formed on the Petri dish. 

### 2.4. Antibiofilm Activity of AgNPs

To assess the ability of nanoparticles to inhibit the initial stages of biofilm formation, the suspensions of the overnight cultures of ESKAPE strains were placed in polystyrene 96-well plates containing Mueller-Hinton broth with 1 MIC AgNPs. Control wells were untreated with AgNPs. The plates were incubated at 37 °C for 24 h. Then, the culture media were discarded to remove non-adherent cells, followed by triple rinsing with phosphate-buffered saline (PBS). To evaluate the volume of biofilm mass attached to the wells, 0.1% (*w*/*v*) gentian violet staining was used. After that, the plates were rinsed and air-dried, and 200 µL of 80% *v*/*v* ethanol was put into each well for the dissolving of connected dye. We measured the optical density (OD) of each well at a wavelength of 595 nm using a Multiskan FC spectrophotometer (Thermo Fisher Scientific, Waltham, MA, USA). To quantify the reduction in biofilm mass ratio in the wells treated with silver nanoparticles, we used the following Formula (3):

(3) $$Reduction\;in\;Biofilm\;Mass\left(\%\right)=\left(\frac{OD\;of\;Treated\;Wells}{OD\;of\;Untreated\;Wells}\right)\times 100\%$$

This Formula calculates the percentage reduction in biofilm mass by comparing the optical density of wells treated with silver nanoparticles to those that were untreated.

For the assessment of the viability of the attached cells under AgNP treatment, we used resazurin assay. Treated and untreated wells with microorganisms were incubated for 24 h. Then, they were washed with PBS and added to 0.05% resazurin solution (200 µL). It was followed by plate incubation at 37 °C for 4 h and the optical density measurements at 570 and 595 nm. With the resazurin solution, medium and microorganisms, but without AgNPs, were used as a positive control. The percentage of cell viability was calculated with a formula proposed by the resazurin manufacturer. Each test was repeated six times, and the OD average was calculated. 

To examine the effect of AgNPs on the viability and biomass volume of the established biofilm, the cultures of ESKAPE microorganisms were incubated in the plates for 48 h before treatment with silver nanoparticles. Then, the non-adherent bacteria were removed from plates, and each well was rinsed in triplicate with PBS. After that, AgNPs diluted with Mueller-Hinton broth at concentrations 10, 20, 30, and 40 µg/mL were added to the wells to treat the established biofilm. The assessment of the biofilm viability and biofilm mass quantity reduction was performed as it was previously described.

### 2.5. The Effect of Silver Nanoparticles on Biofilms Structure

The cell morphology and arrangement of ESKAPE pathogens in biofilms were assessed using SEM. Small glass slides (0.5 × 0.5 cm) were placed in 24-well plate wells containing 2 mL Mueller-Hinton broth with bacterial cells at 5 × 10^5^ CFU/mL concentration and incubated for 48 h at 37 °C. Then, samples were divided into three groups. The first and second groups were added with AgNPs-1 and AgNPs-2 diluted in Muller-Hinton broth at 20 µg/mL concentration. The third group was added to Muller-Hinton broth. After that, all samples were incubated for 24 h. Then, the media were discarded. Samples were washed three times with PBS, fixed with glutaraldehyde 2.5% in 0.1 M phosphate buffer (pH 7.2) for 60 min, dehydrated in ethanol-water mixture with increasing ethanol concentrations (65%, 75%, 85%, 95%, and 100%), and air-dried overnight. Dehydrated specimens were coated with a thin film of silver in a sputter coater. Morphological analysis was performed by the examination of the SEM (Inspect S50-B, FEI, Brno, Czech Republic; accelerating voltage −15 kV) images. 

### 2.6. Statistical Analysis

Statistical significance was determined using an analysis of variance with Graph Pad Prism 9 software, where *p*-value < 0.05 was considered statistically significant. 

## 3. Results

### 3.1. Silver Nanoparticles’ Physical and Chemical Parameters

The structural analyses showed higher surface-area-to-volume ratios in spherical particles (AgNPs-1) compared to cubic shape particles (AgNPs-2) (Table 1). 

The size distribution of the cubic nanoparticles is wide and ranges between 35 and 100 nm (Figure 1). However, a significant part of NPs constitutes particles with a size of 45–75 nm. The spherical particles were with a diameter of 10–60 nm. The bulk of the spherical particles was 35–50 nm. 

Silver ions released from AgNPs were measured at the supernatant on the first and second days after the preparation of the working solution. The colloidal systems containing nanoparticles differed in their ability to release silver ions. The obtained results are presented in Table 2. 

Colloidal solution AgNPs-2 was characterized by a greater ability to form ions in comparison with AgNPs-1, which were slightly more than 10% of the total concentration of silver nanoparticles in the solution. Storing of the AgNPs-2 solution for a day led to a slight increase in the ions’ production. Colloidal solution AgNPs-1 possesses a lower ability to form silver ions in the solution that decreased after storing.

In our study, we measured zeta potential in order to estimate the stability and possible charges on the surfaces of the nanoparticles. Zeta potential measurement of the AgNP-1 and AgNP-2 colloidal solution demonstrated significantly lower results for AgNPs-2 compared to AgNPs-1 (−29.07 ± 0.26 mV, −31.73 ± 0.36 mV, respectively). The evaluation of the silver nanoparticle colloidal solution conductivity revealed significantly higher conductivity of AgNPs-2 than AgNPs-1 (0.101 mS/cm and 0.01 mS/cm, respectively).

### 3.2. Bacterial Strains

We isolated several strains of the ESKAPE pathogens from patients and tested them on sensitivity to antibiotics. For further investigation, we selected one multi-resistant strain of each species. Antibiotic resistance profiles of the selected strains are presented in Table 3. 

### 3.3. Antimicrobial Activity of AgNPs

**Figure 2:**
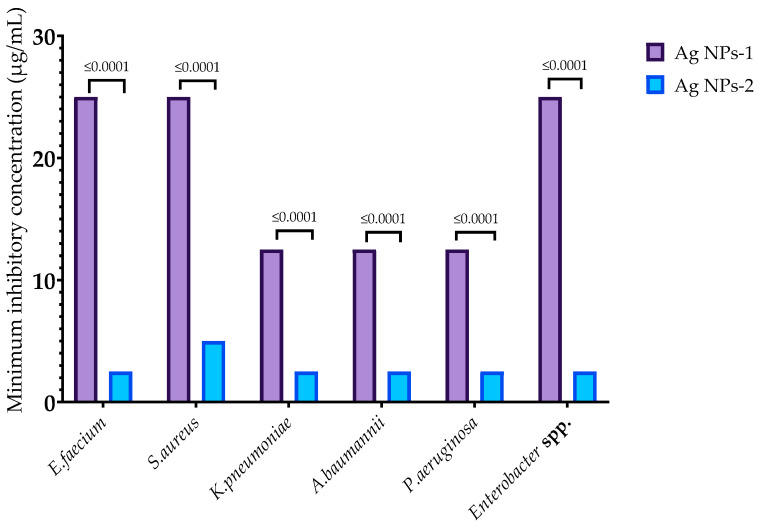
Minimum inhibitory concentration of AgNPs against ESKAPE pathogens.

In this study, AgNPs-1 and AgNPs-2 were used for the investigation of their antibacterial activity against multi-resistant clinical strains of *Enterococcus faecium*, *Staphylococcus aureus*, *Klebsiella pneumoniae*, *Acinetobacter baumannii*, *Pseudomonas aeruginosa*, and *Enterobacter* spp. Both samples were effective against all pathogens. MIC of examined nanoparticles varied from 2.5 to 25 µg/mL (Figure 2). MICs of AgNPs-2 were significantly lower than for AgNPs-1. However, there was no strict correlation between the NP activity and the structure of bacteria cell walls (Gram-negative or Gram-positive germs).

**Figure 3:**
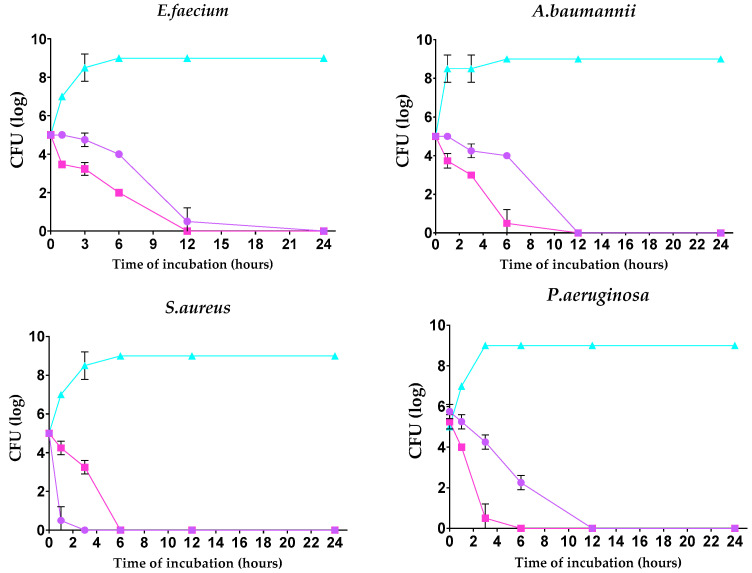

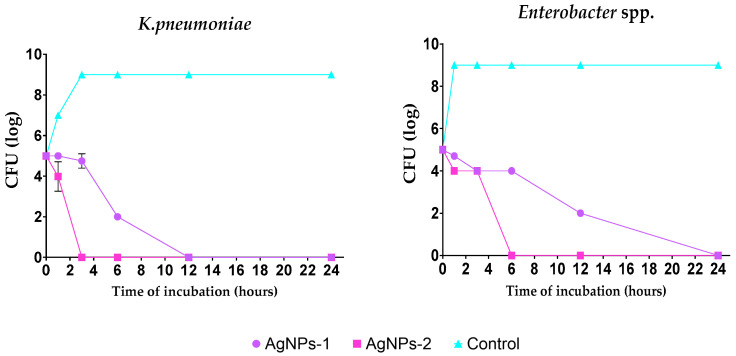
Time-dependent bactericidal activity of the tested silver nanoparticles against ESKAPE pathogens (log CFU/h).

The kill kinetics profile of AgNPs-1 and AgNPs-2 demonstrates a gradual drop in the viable cells’ numbers over the experimental time. The number of bacteria cells in all samples decreased to 0 log CFU/mL within incubation time. However, the speed of bacteria response to AgNPs-1 and AgNPs-2 action was various (Figure 3). In most cases (except *S. aureus*), AgNPs-2 killed bacteria rapidly compared to AgNPs-1. Almost all Gram-negative bacteria were killed with AgNPs-2 by 6 h of incubation.

### 3.4. Antibiofilm Activity of AgNPs

**Figure 4:**
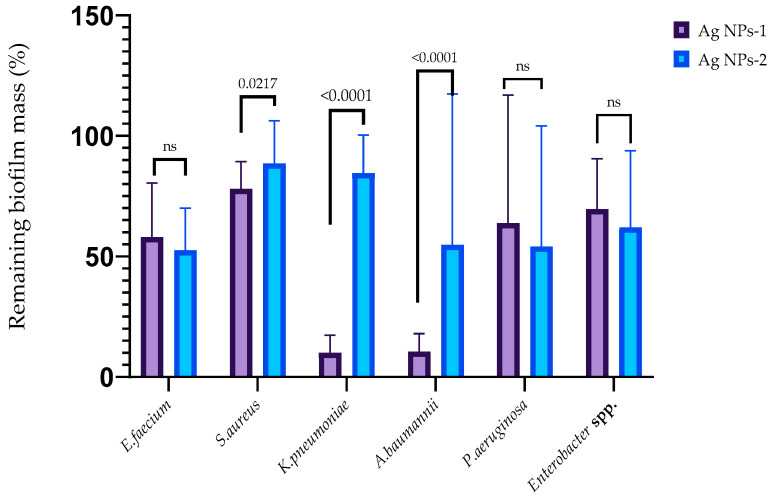

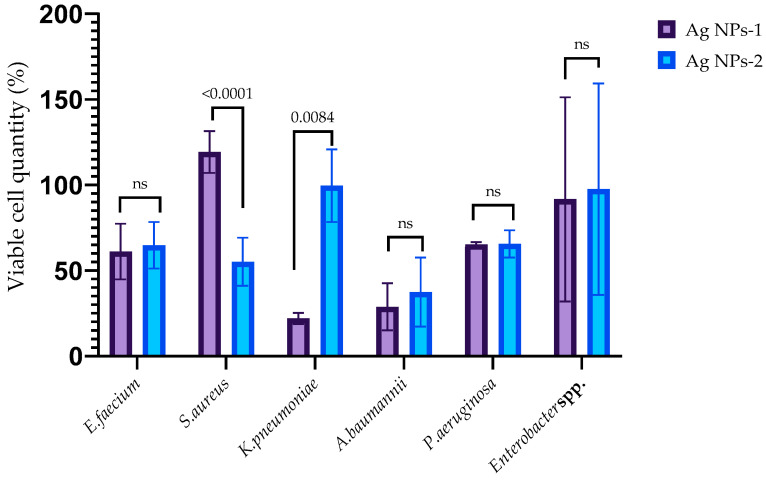
The anti-adhesion activity of AgNPs against ESKAPE pathogens (*p*-value is above the bars, ns—nonsignificant difference).

The ability of AgNPs-1 and AgNPs-2 to inhibit the early stages of biofilm formation is demonstrated in Figure 4. It revealed the various sensitivity of the ESKAPE pathogens to silver nanoparticles as well as the effectiveness of different kinds of AgNPs-1 and AgNPs-2. Both types of AgNPs at a concentration equal to 1 MIC (2.5 to 25 µg/mL, respectively, to AgNPs 2 and AgNPs 1) inhibited the formation of the biofilm mass to varying degrees. AgNPs-1 at concentration 1 MIC more effectively prevented the accumulation of the biofilm mass of *S. aureus*, *K. pneumoniae*, and *A. baumannii* (*p* ≤ 0.05) than AgNPs-2. The pattern of the AgNPs’ action on the biofilm cell viability in most cases replicated the effect of silver nanoparticles on the biofilm mass with one exception. AgNPs-2 more effectively reduced biofilm cell viability of *S. aureus* than biofilm mass in comparison with AgNPs-1. 

**Figure 5:**
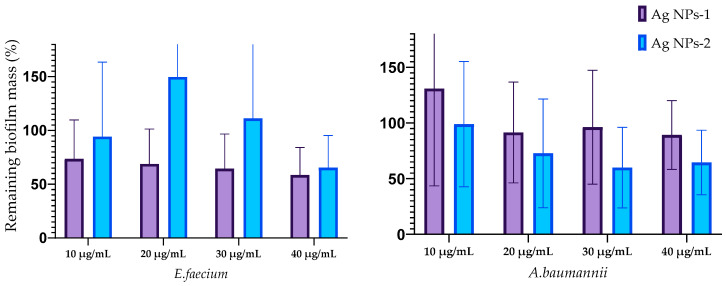

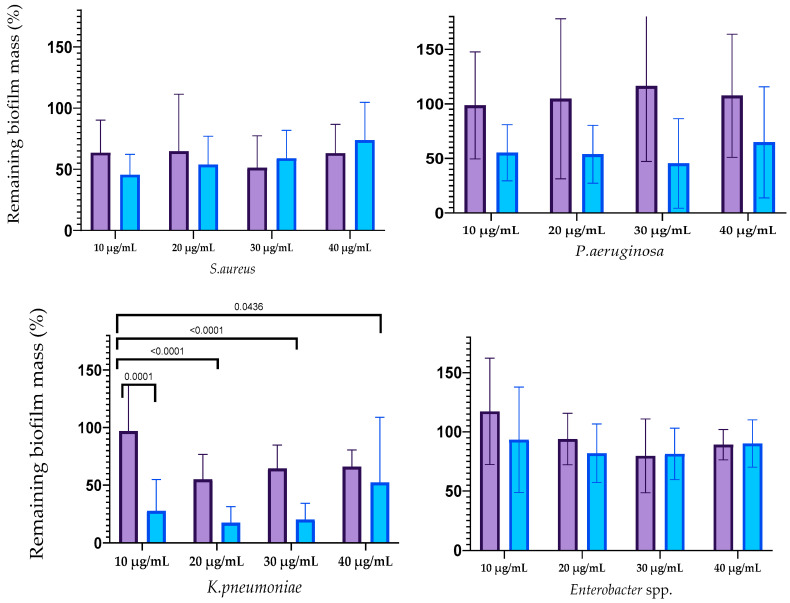
Reduction in the biofilm mass volume of the ESKAPE pathogens treated with AgNPs.

The influence of silver nanoparticles on mature biofilm mass is reflected in Figure 5. The destruction of biofilms formed with *E. faecium* was caused by AgNPs-1 at all concentrations and AgNPs-2 at a concentration of 40 µg/mL. The biofilms formed by *S. aureus* were destroyed with AgNP-1 and AgNPs-2 at all concentrations. The efficacy of AgNPs-1 and AgNPs-2 in disrupting mature biofilm mass varied among the tested Gram-negative bacteria. Specifically, AgNPs-1, at concentrations ranging from 20–40 µg/mL, was effective only against the biofilm formed by *K. pneumoniae*. In contrast, AgNPs-2 demonstrated a variable degree of effectiveness in disrupting the biofilms of all tested Gram-negative microbes, including *A. baumannii*, *P. aeruginosa*, *K. pneumoniae*, and *Enterobacter* spp. However, the significant difference between AgNPs-1 and AgNPs-2 activity was detected only against *K. pneumoniae* biofilm.

**Figure 6:**
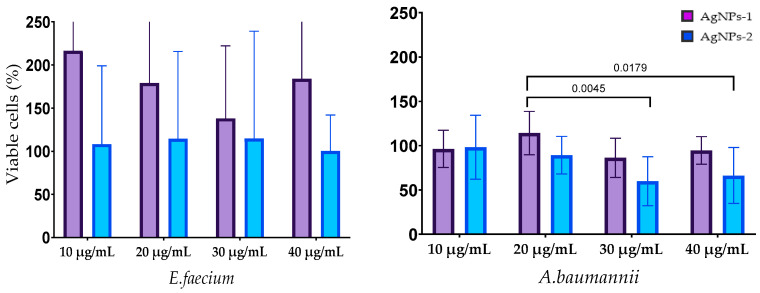

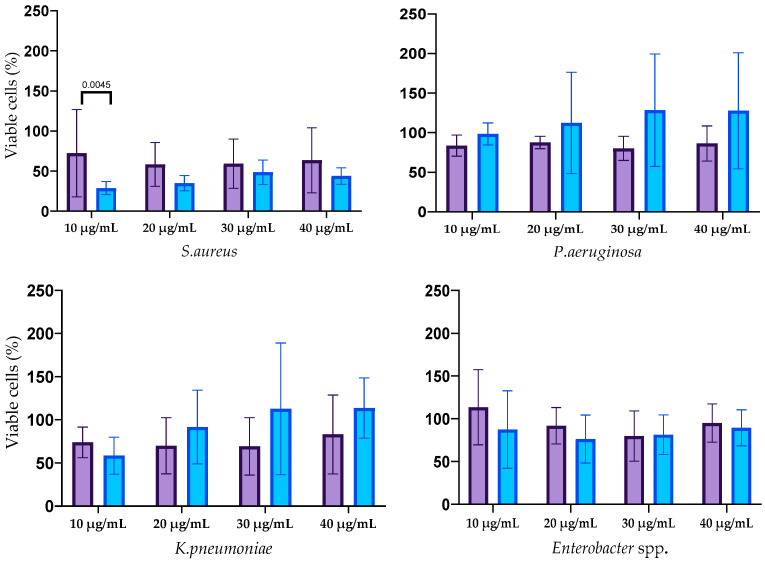
The influence of AgNPs-1 and AgNPs-2 on cell viability of mature biofilms formed with ESKAPE pathogens.

The results of the biofilm cells’ viability after silver nanoparticle treatment are shown in Figure 6. The effect of AgNPs on the biofilm cells of E. faecium was not revealed. Cell viability in mature biofilms formed by *S. aureus* was decreased by treatment with both types of AgNPs at all concentrations with a statistically significant difference at concentration 10 µg/mL. AgNPs-2 caused the statistically reliable decline of the *A. baumannii* biofilm viability at a concentration of 30–40 µg/mL. There was a slight decrease in the viable cell count in the biofilm formed with *K. pneumoniae*, *P. aeruginosa,* and *Enterobacter* spp. At the same time, the ANOVA (one way) did not show a significant difference between AgNPs-1 and AgNPs-2 activity. 

### 3.5. Effect of Silver Nanoparticles on the Biofilms Structure

**Figure 7:**
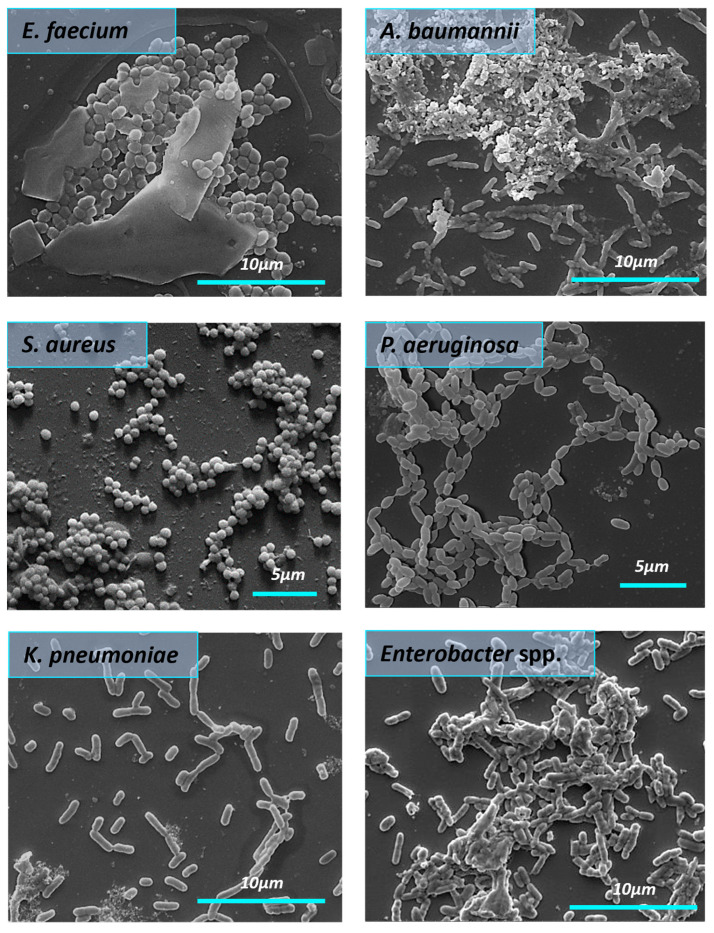
The morphology of the mature biofilms formed by ESKAPE pathogens (SEM). The red arrows demonstrate *A. baumannii* pili.

All bacteria in the control group formed the structured biofilms. *S. aureus* biofilms were dense and highly hydrated, with clusters of bacterial cells enclosed in a matrix composed of exopolysaccharides. In the control group (Figure 7), we observed the *E. faecium* biofilm biomass as a monolayer with visible 3D architecture. *E. faecium* biofilm was embedded into the EPS wrapping. The biofilm formed by *A. baumannii* in the control sample was characterized by the presence of pili or cellular filaments between bacterial cells. A similar picture was found in the case of the *K. pneumoniae* where biofilm was presented as cell-associated bacterial clusters. SEM micrograph of the *Enterobacter* spp. bacteria in the control group showed the aggregation and cohesion of the cells with visible intimate contact between bacteria and the surface. Biofilm architecture of untreated *P. aeruginosa* biofilm appeared as aggregated rod chains by pili.

**Figure 8:**
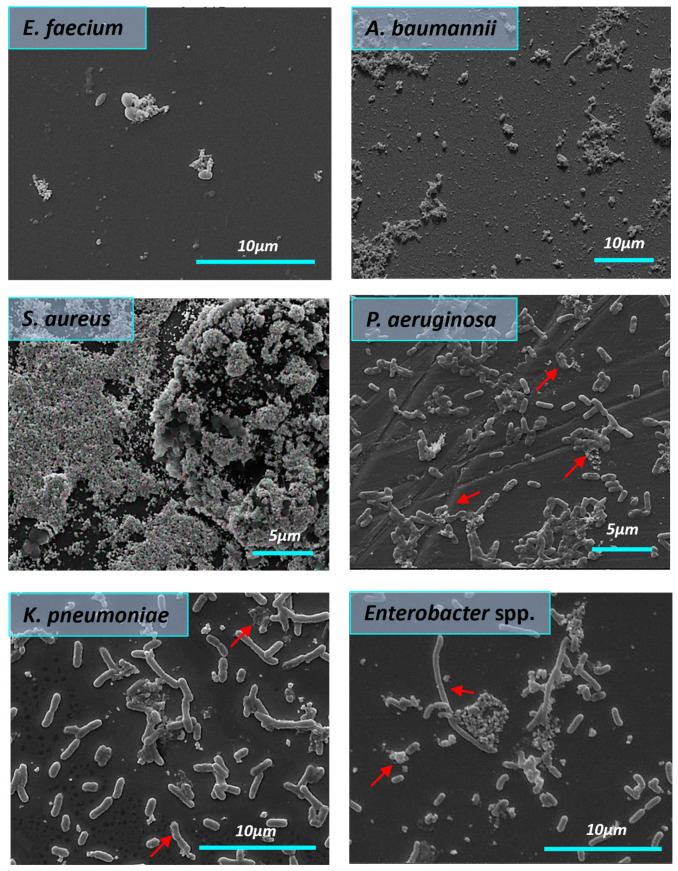
The influence of AgNPs-1 on the structure of mature biofilms formed by ESKAPE pathogens (SEM). The red arrows demonstrate cell changes after AgNPs-1 treatment.

The influence of AgNPs-1 on the structure of the biofilms formed by ESKAPE pathogens is shown in Figure 8. There is a clear trend of AgNPs-1 effect on bacterial biofilm. It was found that AgNPs-1 destroyed the biofilm formed by *S. aureus*. SEM findings revealed the disrupted biofilm structure. There were only a few scattered bacteria adhered to the glass surface. A decrease in *E. faecium* cell numbers was observed after treatment with AgNPs-1 as well because of cell death. Morphological changes in the cell wall of *E. faecium* were indicated using SEM analysis. Figure 8 clearly shows the absence of *A. baumannii* biofilm after treatment with AgNPs-1. AgNPs-1 treatment caused the changes in *A. baumannii* cell morphology. The cell surface, conformation, and size are drastically affected. *K. pneumoniae* cell clusters lose their pili after cultivation with AgNPs-1. SEM micrographs revealed that some *Enterobacter* spp. Cells treated with AgNPs-1 were more elongated compared to the control strain (red arrow). There was a lower number of adhered cells. *Enterobacter* spp. cells were arranged in the form of aggregates or simply as individualized cells without slimy material in their vicinity. *P. aeruginosa* cells in the presence of AgNPs-1 showed a lawn of separated and aggregated bacteria with fewer rod chains as well. 

**Figure 9:**
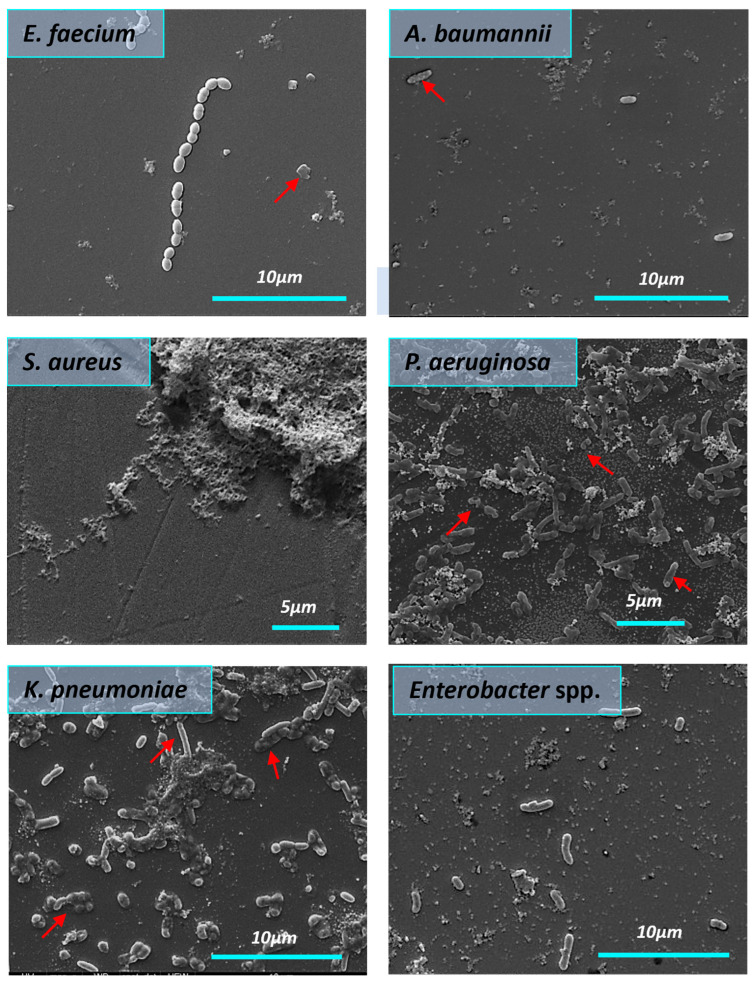
The influence of AgNPs-2 on the structure of mature biofilms formed by ESKAPE pathogens (SEM). The red arrows demonstrate cell changes after AgNP treatment.

Figure 9 demonstrates the effectiveness of AgNPs-2 against almost all tested strains. SEM images showed abnormalities of the cell membrane. Membrane disintegration and perforation were detected, as well as shrinkage and leakage of intracellular cytoplasmic content (marked with a red arrow). AgNPs-2 destroyed the mature biofilm formed by *S. aureus* and *E. faecium*. The last one formed distinct cell aggregates under AgNPs-2 action. AgNPs-2 altered *K. pneumoniae* cell morphology. There was severe disruption of the cell surface, cytoplasmic leakage, and lysed cells. AgNPs-2 demonstrates a severe effect on *Enterobacter* spp. biofilm. There were signs of cell wall damage under AgNPs-2 treatment. *P. aeruginosa* cells were ruptured, shrunken, and lost cellular components under the influence of AgNPs-2. 

## 4. Discussion

ESKAPE pathogens are considered the most dangerous germs due to their antimicrobial resistance mechanisms and accelerated ability to form biofilm [33,34]. Thus, there is an urgent need for new, effective antimicrobial drug development to combat these microorganisms [34]. Nanomaterials are promising alternatives to conventional antibiotics [12]. Several publications report the antibacterial effectiveness of silver nanoparticles against different bacteria [35], fungi [36], and viruses [37]. However, the results obtained by different authors are often controversial and have low reproducibility [38]. Besides this variety of the applied study design, nanoparticle synthesis, and examined microorganisms make cross-interpretation between these studies complicated. Moreover, there is no data about the investigation of the silver nanoparticles’ action on the whole list of ESKAPE pathogens in the frame of one research. Due to these, there is a need to investigate and compare the sensitivity of these pathogens to silver nanoparticles. 

### 4.1. Nanoparticle Size and Shape

In this study, the antimicrobial activity of two types of AgNPs was evaluated against multi-resistant clinical ESKAPE isolates in planktonic and biofilm forms. We used AgNPs prepared using the chemical reduction method with the capping substance PVP, but with some differences in preparation techniques. It leads to the formation of different shapes and sizes of nanoparticles. AgNPs-1 were round-shaped with an average size of 44.6 ± 3.22 nm and a particle size distribution of 10–60 nm. AgNPs-2 were cube-shaped with an average length of 70 ± 35 nm and particle size in the range from 35 to 100 nm. Several reports have indicated the significant impact of the AgNPs’ size and shape on their antimicrobial activity. It was shown that smaller and round-shaped particles [21] demonstrate higher antimicrobial activity. Researchers attribute the higher antibacterial activity of these NPs to their greater release of silver ions [39]. Contrary to expectations, this study found that bigger cube-shaped particles (AgNPs-2) exhibited five times higher antimicrobial effectiveness compared to smaller round-shaped ones (AgNPs-1) against all tested microorganisms. MICs varied from 2.5 to 5.0 and from 12.5 to 25 µg/mL, respectively, to AgNPs-2 and AgNPs-1. Aside from this, AgNPs-2 also demonstrated a higher release of the Ag^+^ ions, and these findings are not consistent with previous research. Future studies will be required to consider these variables.

### 4.2. Antibacterial Mechanisms

Researchers suggest three main mechanisms for the antimicrobial activity of AgNPs such as the formation of reactive oxygen species (ROS), the ion release process, and non-oxidative mechanisms [39]. The effectiveness of a non-oxidative mechanism or contact killing depends on the contact surface area and surface-to-volume ratio (SA:V). Some authors demonstrated that a high surface area allows an enhanced antimicrobial effect [40]. The charge status of NPs also has a significant impact on their affinity to bacterial cell membranes and their subsequent entry into the bacteria [41]. The current study found that AgNPs-1 possesses a higher contact surface area and a zeta potential below −30 mV, which indicates its high stability. Several publications indicate that positively surface-charged AgNPs provide more effective electrostatic interaction with the negatively charged bacterial cell than negatively charged NPs, which ensures their antibacterial activity [42]. Despite all this, AgNPs-2 exhibited higher antibacterial activity against a planktonic form of the examined bacteria compared to AgNPs-1. We suggest the proper polymeric barrier effectively prevents aggregation and provides better antimicrobial activity of AgNPs-2. The SEM images confirmed the ability of the tested AgNPs to attach to the bacteria cell, resulting in disruption of the cell membrane. Probably, the non-oxidative mechanism takes place as a mode of AgNP antimicrobial activity.

Differences in AgNPs-1 and AgNPs-2 antibacterial activity may be attributed to sharp edges of the nanoparticles. Previous studies noted that the higher antibacterial properties of the cubic nanostructures could be associated with an increase in the strength of electric field intensity, higher surface energy, and more highly reactive facets [43,44,45]. As a result, these nanostructures have stronger interaction with bacterial cell membranes, which leads to rapid interactions with oxygen-containing groups of bacterial lipopolysaccharides and induces cell membrane damage [46]. Mammari N. et al. [42] suggested that the generation of abiotic ROS is the leading mechanism by which AgNPs target cell wall components, resulting in disordered states of the fatty-acid tail of the phospholipid phosphatidylethanolamine (PE). Considering these two suggestions, we assume that AgNPs-2’s antimicrobial activity is based mostly on ROS production. 

This study has been unable to demonstrate the stronger antibacterial effect of AgNPs against Gram-negative bacteria than Gram-positive bacteria. Our results are consistent with data obtained in [44]. Obviously, we cannot narrow down the understanding of the AgNPs’ antibacterial action only based on their Gram-staining characteristics. These results support the idea of Ferreyra A.P. [47], who supposes the individual combination of AgNP antibacterial mechanisms for every species. 

Another important aspect of this work was to analyze the minimum time necessary to reach bactericidal effect. Several studies have shown that the effect of AgNPs is time-dependent [25,36]. Different reports demonstrated the entire mortality in four [44] or eight hours of incubation at MIC [48]. The current study found that AgNPs-2 inhibited the growth and reproduction of the most number of isolates faster than AgNPs-1. In general, these data align with previous results and support the hypothesis that the antimicrobial activity of AgNPs might be linked to NP’s particular features, such as zeta potential and level of ion release. 

### 4.3. Biofilm Formation

Despite microbes that exist predominately within biofilms in natural and clinical settings, the biggest part of AgNPs’ antimicrobial activity investigation is focused mostly on the planktonic form. Biofilm resistance to antimicrobial treatment is 100–1000 times higher compared to planktonic cells [49]. In our investigation, we evaluated the ability of AgNPs to prevent cellular attachment and destroy mature biofilm. Both types of examined NPs inhibited the attachment of the ESKAPE pathogens at a concentration equal to 1.0 MIC, with significantly higher effectiveness of AgNPs-1 against *S. aureus*, *K. pneumoniae*, and *A. baumannii* (*p* ≤ 0.05). It confirms the effectiveness of AgNPs as antiadhesive compounds at comparatively low concentrations.

After primary attachment, bacterial cells start to divide and produce EPS as a main component of a biofilm matrix [49]. We used a comparatively lower concentration of AgNPs (10–40 µg/mL) in the treatment of the mature biofilms than what was reported earlier [50]. Both types of examined AgNPs destroyed the mature biofilms formed by all tested ESKAPE strains. There was a decrease in mature biofilm mass of all tested microbes treated with AgNPs-1 and AgNPs-2. The SEM images confirmed the data obtained with gentian violet and resazurin assays. Both types of nanoparticles demonstrated effectiveness against all tested Gram-positive strains and different degrees of antibiofilm activity against Gram-negative germs. Statistically reliable, the higher effectiveness of AgNPs-2 in decreasing *K. pneumoniae* biofilm mass was detected in comparison to AgNPs-1 according to the gentian violet assay. AgNPs-2 exhibited a more pronounced reduction in cell viability compared to AgNPs-1 when tested against mature biofilms formed by *S. aureus* and *A. baumannii*, achieving reductions of up to 28% and 59%, respectively. The underlying reasons for the variations in species sensitivity to AgNPs remain unclear. It is noteworthy that the anti-biofilm activity of AgNPs is due to their ability to destroy the extracellular polymeric substance (EPS) matrix and induce cell death [51]. A recent systematic literature review highlighted that the EPS matrix comprises exopolysaccharides, extracellular DNA (e DNA), lipids, proteins, and other macromolecules, with its composition being unique for each ESKAPE pathogen [52]. SEM analyses revealed the disruption of the mature biofilm structure and different changes in cell morphology under the AgNP treatment. The variation in biofilm formation among ESKAPE pathogens is related to the differences in species-specific components within the EPS matrix. These findings underscore the need for further research to elucidate the species-specific mechanisms through which nanoparticles disrupt biofilms. 

Based on the findings reported here, we can assume that some unmeasured variables could account for some aspects of the results. Consequently, key issues of the AgNP’s influence on interactions between host surfaces and microbes in the formation of biofilms need to be resolved in the following investigations to ensure successful clinical applications.

## 5. Conclusions

In conclusion, this study demonstrates the potential of silver nanoparticles (AgNPs) as effective antimicrobial agents against clinical strains of ESKAPE pathogens, including multidrug-resistant bacteria. The results indicate that the physical and chemical properties of AgNPs, such as size, shape, and surface charge, play a crucial role in determining their antimicrobial and antibiofilm activities. The cubic-shaped AgNPs exhibited superior antimicrobial efficacy compared to spherical AgNPs. Both types of AgNPs showed the ability to inhibit biofilm formation and disrupt established biofilms, leading to membrane damage and reduced viability of the bacteria. These findings support the potential of AgNPs as a promising alternative to conventional antibiotics for combating biofilm-related infections caused by ESKAPE pathogens. Further research is warranted to elucidate the underlying mechanisms of AgNPs’ action, optimize their formulation, and evaluate their safety and efficacy in preclinical and clinical settings. Harnessing the antimicrobial potential of nanomaterials like AgNPs offers new possibilities for addressing the urgent global challenge of antibiotic resistance and improving the management of infectious diseases.

## Figures and Tables

**Table 1:** Morphology parameters of the AgNPs.

AgNPs-1		AgNPs-2	
Average radius (r) ± SD, nm	Average SA/V ± SD	Average length (a) ± SD, nm	Average SA/V ± SD
22.3 ± 3.22	0.14 ± 0.021	70 ± 35 *	0.1 ± 0.03 *

* Significant difference with AgNPs-1 group, SA/V—surface area to volume ratio, SD—standard deviation.

**Table 2:** Silver ions release from AgNPs.

	AgNPs-1		AgNPs-2	
	µg/mL	%	µg/mL	%
Silver content in the working solution	129.0	-	194.7	-
The content of silver ions in the supernatant on the first day after preparation	2.3	1.7	19.7	10.1
The content of silver ions in the supernatant on the second day after preparation	1.1	0.85	25.7	13.1

**Table 3:** Resistance profiles of isolated microorganisms.

Strain	Profile of Strains Sensitivity to Antibiotics							
	Amo	Imi	Van	Gat	Cep	Cet	Ami	Azi
*E. faecium*	R	R	S	S	R	S	S	R
*S. aureus*	R	R	R	S	R	R	R	R
*K. pneumoniae*	R	S	-	R	R	R	S	R
*A. baumannii*	R	R	-	S	R	R	S	R
*P. aeruginosa*	R	S	-	R	R	R	R	S
*Enterobacter* spp.	R	R	-	-	R	S	S	R

R—resistant, S—sensitive, Amo—amoxicillin, Imi—imipenem, Van—vancomycin, Gat—gatifloxacin, Cep—cefepime, Cet—cefotaximum, Ami—amikacin, Azi—azithromycin.

## Data Availability

The data are not accessible because they are part of the ongoing project. Data should be requested in correspondence authors.

## References

[1] Ventola C.L. (2015). The antibiotic resistance crisis: Part 1: Causes and threats. Pharm. Ther..

[2] World Health Organization (2018). WHO Report on Surveillance of Antibiotic Consumption.

[3] E Marturano J., Lowery T.J. (2019). ESKAPE Pathogens in Bloodstream Infections Are Associated With Higher Cost and Mortality but Can Be Predicted Using Diagnoses Upon Admission. Open Forum Infect. Dis..

[4] Santajit S., Indrawattana N. (2016). Mechanisms of Antimicrobial Resistance in ESKAPE Pathogens. Biomed. Res. Int..

[5] Patil A., Banerji R., Kanojiya P., Saroj S.D. (2021). Foodborne ESKAPE Biofilms and Antimicrobial Resistance: Lessons Learned from Clinical Isolates. Pathog. Glob. Health.

[6] Pinto R.M., Soares F.A., Reis S., Nunes C., Van Dijck P. (2020). Innovative Strategies Toward the Disassembly of the EPS Matrix in Bacterial Biofilms. Front. Microbiol..

[7] Murray E.J., Dubern J.-F., Chan W.C., Chhabra S.R., Williams P. (2022). Pseudomonas aeruginosa PQS quorum-sensing system inhibitor with anti-staphylococcal activity sensitizes polymicrobial biofilms to tobramycin. Cell Chem. Biol..

[8] Gebreyohannes G., Nyerere A., Bii C., Sbhatu D.B. (2019). Challenges of intervention, treatment, and antibiotic resistance of biofilm-forming microorganisms. Heliyon.

[9] Mulani M.S., Kamble E.E., Kumkar S.N., Tawre M.S., Pardesi K.R. (2019). Emerging Strategies to Combat ESKAPE Pathogens in the Era of Antimicrobial Resistance: A Review. Front. Microbiol..

[10] Abebe G.M. (2020). The Role of Bacterial Biofilm in Antibiotic Resistance and Food Contamination. Int. J. Microbiol..

[11] Bruna T., Maldonado-Bravo F., Jara P., Caro N. (2021). Silver Nanoparticles and Their Antibacterial Applications. Int. J. Mol. Sci..

[12] Hemeg H.A. (2017). Nanomaterials for alternative antibacterial therapy. Int. J. Nanomed..

[13] Bhatia D., Mittal A., Malik D.K. (2021). Antimicrobial potential and in vitro cytotoxicity study of polyvinyl pyrollidone-stabilised silver nanoparticles synthesised from Lysinibacillus boronitolerans. IET Nanobiotechnol..

[14] Klink M.J., Laloo N., Taka A.L., Pakade V.E., Monapathi M.E., Modise J.S. (2022). Synthesis, Characterization and Antimicrobial Activity of Zinc Oxide Nanoparticles against Selected Waterborne Bacterial and Yeast Pathogens. Molecules.

[15] Kamel S.M., Elgobashy S.F., Omara R.I., Derbalah A.S., Abdelfatah M., El-Shaer A., Al-Askar A.A., Abdelkhalek A., Abd-Elsalam K.A., Essa T. (2022). Antifungal Activity of Copper Oxide Nanoparticles against Root Rot Disease in Cucumber. J. Fungi.

[16] Žalnėravičius R., Pakštas V., Grincienė G., Klimas V., Paškevičius A., Timmo K., Kauk-Kuusik M., Franckevičius M., Niaura G., Talaikis M. (2023). Antimicrobial particles based on Cu2ZnSnS4 monograins. Colloids Surf. B Biointerfaces.

[17] Žalnėravičius R., Klimas V., Paškevičius A., Grincienė G., Karpicz R., Jagminas A., Ramanavičius A. (2021). Highly efficient antimicrobial agents based on sulphur-enriched, hydrophilic MoS2 nano/microparticles and heterostructured Pd/MoS2/Ti coatings. J. Colloid Interface Sci..

[18] Mateo E.M., Jiménez M. (2022). Silver Nanoparticle-Based Therapy: Can It Be Useful to Combat Multi-Drug Resistant Bacteria?. Antibiotics.

[19] Khatoon U.T., Rao G.N., Mohan M.K., Ramanaviciene A., Ramanavicius A. (2018). Comparative study of Antifungal Activity of Silver and Gold Nanoparticles Synthesized by Facile Chemical Approach. J. Environ. Chem. Eng..

[20] Khatoon U.T., Rao G.N., Mohan K.M., Ramanaviciene A., Ramanavicius A. (2017). Antibacterial and antifungal activity of silver nanospheres synthesized by tri-sodium citrate assisted chemical approach. Vacuum.

[21] Alshareef A., Laird K. (2017). Shape-dependent antibacterial activity of silver nanoparticles on Escherichia coli and Enterococcus faecium bacterium. Appl. Surf. Sci..

[22] McNeilly O., Mann R., Hamidian M., Gunawan C. (2021). Emerging Concern for Silver Nanoparticle Resistance in Acinetobacter baumannii and Other Bacteria. Front. Microbiol..

[23] Dheyab M.A., Aziz A.A., Oladzadabbasabadi N., Alsaedi A., Braim F.S., Jameel M.S., Ramizy A., Alrosan M., Almajwal A.M. (2023). Comparative Analysis of Stable Gold Nanoparticles Synthesized Using Sonochemical and Reduction Methods for Antibacterial Activity. Molecules.

[24] Xu L., Wang Y.-Y., Huang J., Chen C.-Y., Wang Z.-X., Xie H. (2020). Silver nanoparticles: Synthesis, medical applications and biosafety. Theranostics.

[25] Javed R., Zia M., Naz S., Aisida S.O., Ain N.U., Ao Q. (2020). Role of Capping Agents in the Application of Nanoparticles in Biomedicine and Environmental Remediation: Recent Trends and Future Prospects. J. Nanobiotechnol..

[26] Zein R., Alghoraibi I., Soukkarieh C., Ismail M.T., Alahmad A. (2022). Influence of Polyvinylpyrrolidone Concentration on Properties and Anti-Bacterial Activity of Green Synthesized Silver Nanoparticles. Micromachines.

[27] Lee N.-Y., Ko W.-C., Hsueh P.-R. (2019). Nanoparticles in the treatment of infections caused by multidrug-resistant organisms. Front. Pharmacol..

[28] Khan M.H., Unnikrishnan S., Ramalingam K. (2019). Bactericidal potential of silver-tolerant bacteria derived silver nanoparticles against multi drug resistant ESKAPE pathogens. Biocatal. Agric. Biotechnol..

[29] Musthafa M., Gobianand K., Manohar M. (2020). Anti-ESKAPE activity of green synthesized silver nanoparticles from Picrorhiza Kurroa royle ex benth. Int. J. Pharm. Sci. Res..

[30] Myronov P., Bugaiov V., Holubnycha V., Sikora V., Deineka V., Lyndin M., Opanasyuk A., Romaniuk A., Pogorielov M. (2020). Low-frequency ultrasound increase efectiveness of silver nanoparticles in a purulent wound model. Biomed. Eng. Lett..

[31] Krolicka A., Banasiuk R., Frackowiak J.E., Krychowiak M., Matuszewska M., Kawiak A., Ziabka M., Lendzion-Bielun Z., Narajczyk M. (2016). Synthesis of antimicrobial silver nanoparticles through a photomediated reaction in an aqueous environment. Int. J. Nanomedicin..

[32] Limbago B. (2001). M100-S11, Performance standards for antimicrobial susceptibility testing. Clin. Microbiol. Newsl..

[33] Ma Y.X., Wang C.Y., Li Y.Y., Li J., Wan Q.Q., Chen J.H., Tay F.R., Niu L.N. (2020). Considerations and Caveats in Combating ESKAPE Pathogens against Nosocomial Infections. Adv. Sci..

[34] Kamaruzzaman N.F., Tan L.P., Hamdan R.H., Choong S.S., Wong W.K., Gibson A.J., Chivu A., Pina M.D.F. (2019). Antimicrobial Polymers: The Potential Replacement of Existing Antibiotics?. Int. J. Mol. Sci..

[35] Loo Y.Y., Rukayadi Y. (2018). In Vitro Antimicrobial Activity of Green Synthesized Silver Nanoparticles Against Selected Gram-negative Foodborne Pathogens. Front. Microbiol..

[36] Elgorban A.M., El-Samawaty A.E.R.M., Yassin M.A., Sayed S.R., Adil S.F., Elhindi K.M., Bakri M., Khan M. (2016). Antifungal Silver Nanoparticles: Synthesis, Characterization and Biological Evaluation. Biotechnol. Biotechnol. Equip..

[37] Galdiero S., Falanga A., Vitiello M., Cantisani M., Marra V., Galdiero M. (2011). Silver Nanoparticles as Potential Antiviral Agents. Molecules.

[38] Quintero-Quiroz C., Acevedo N., Zapata-Giraldo J., Botero L.E., Quintero J., Zárate-Trivinõ D., Saldarriaga J., Pérez V.Z. (2019). Optimization of Silver Nanoparticle Synthesis by Chemical Reduction and Evaluation of Its Antimicrobial and Toxic Activity. Biomater. Res..

[39] Fernandes M., González-Ballesteros N., da Costa A., Machado R., Gomes A.C., Rodríguez-Argüelles M.C. (2023). Antimicrobial and Anti-Biofilm Activity of Silver Nanoparticles Biosynthesized with Cystoseira Algae Extracts. J. Biol. Inorg. Chem..

[40] Sayed F.A.Z., Eissa N.G., Shen Y., Hunstad D.A., Wooley K.L., Elsabahy M. (2022). Morphologic Design of Nanostructures for Enhanced Antimicrobial Activity. J. Nanobiotechnol..

[41] Yuan Q., Xiao R., Afolabi M., Bomma M., Xiao Z. (2023). Evaluation of Antibacterial Activity of Selenium Nanoparticles against Food-Borne Pathogens. Microorganisms.

[42] Mammari N., Lamouroux E., Boudier A., Duval R.E. (2022). Current Knowledge on the Oxidative-Stress-Mediated Antimicrobial Properties of Metal-Based Nanoparticles. Microorganisms.

[43] Tang S., Zheng J. (2018). Antibacterial Activity of Silver Nanoparticles: Structural Effects. Adv. Healthc. Mater..

[44] González-Fernández S., Lozano-Iturbe V., García B., Andrés L.J., Menéndez M.F., Rodríguez D., Vazquez F., Vazquez F., Martín C., Quirós L.M. (2020). Antibacterial Effect of Silver Nanorings. BMC Microbiol..

[45] Cheon J.Y., Kim S.J., Rhee Y.H., Kwon O.H., Park W.H. (2019). Shape-Dependent Antimicrobial Activities of Silver Nanoparticles. Int. J. Nanomed..

[46] Menichetti A., Mavridi-Printezi A., Mordini D., Montalti M. (2023). Effect of Size, Shape and Surface Functionalization on the Antibacterial Activity of Silver Nanoparticles. J. Funct. Biomater..

[47] Ferreyra A.P., Gonçalves S. (2019). Studies on interaction of green silver nanoparticles with whole bacteria by surface characterization techniques. BBA-Biomembr..

[48] Das B., Kumar S. (2017). Green synthesized silver nanoparticles destroy multidrug resistant bacteria via reactive oxygen species mediated membrane damage. Arab. J. Chem..

[49] Anees Ahmad S., Sachi Das S., Khatoon A., Tahir Ansari M., Afzal M., Saquib Hasnain M., Kumar Nayak A. (2020). Bactericidal Activity of Silver Nanoparticles: A Mechanistic Review. Mater. Sci. Energy Technol..

[50] Samrot A.V., Mohamed A.A., Faradjeva E., Jie L.S., Sze C.H., Arif A., Sean T.C., Michael E.N., Mun C.Y., Qi N.X. (2021). Mechanisms and Impact of Biofilms and Targeting of Biofilms Using Bioactive Compounds—A Review. Medicina.

[51] Coriolano D.d.L., de Souza J.B., Bueno E.V., Medeiros S.M.d.F.R.d.S., Cavalcanti I.D.L., Cavalcanti I.M.F. (2021). Antibacterial and antibiofilm potential of silver nanoparticles against antibiotic-sensitive and multidrug-resistant Pseudomonas aeruginosa strains. Braz. J. Microbiol..

[52] Mukherjee A., Bose S., Shaoo A., Das S.K. (2023). Nanotechnology based therapeutic approaches: An advanced strategy to target the biofilm of ESKAPE pathogens. Mater. Adv..

